# Management of Periprosthetic Proximal Humerus Fractures Following Shoulder Arthroplasty: A Systematic Review

**DOI:** 10.7759/cureus.112556

**Published:** 2026-07-13

**Authors:** Kashif Memon, Manahil Awan, Shahzad Ahmad, Shashwat Shetty

**Affiliations:** 1 Trauma and Orthopedics, Queen Elizabeth Hospital Birmingham, Birmingham, GBR; 2 Trauma and Orthopedics, Heartlands Hospital, Birmingham, GBR; 3 Surgery, Mater Private Hospital, Dublin, IRL; 4 Orthopedics, Hillingdon Hospital, Uxbridge, GBR

**Keywords:** cofield classification, fracture management, orif, periprosthetic humeral fracture, revision arthroplasty, shoulder arthroplasty, worland classification

## Abstract

Periprosthetic proximal humeral fractures following shoulder arthroplasty are rare but present significant clinical challenges due to variable fracture patterns, stem stability, and patient factors. This systematic review aimed to summarize contemporary evidence on management strategies, including nonoperative treatment, open reduction and internal fixation (ORIF), and revision arthroplasty, and to evaluate outcomes, complications, and factors influencing fracture healing. A comprehensive search of PubMed, Embase, Scopus, and the Cochrane Library identified eight studies meeting the inclusion criteria, encompassing 210 patients with fractures classified using the Cofield or Worland systems. ORIF was the most commonly reported intervention, achieving union rates of up to 93%, while nonoperative management was associated with higher nonunion and malunion rates. Revision arthroplasty provided moderate union rates (~84%) but carried higher complication risks. Successful outcomes were linked to humeral stem stability, fracture displacement, and individualized, fracture-specific management. Current evidence highlights the need for standardized classification systems, prospective multicenter studies, and evaluation of novel fixation techniques to improve union rates and functional recovery in this complex patient population.

## Introduction and background

Periprosthetic humeral fractures are uncommon but challenging complications following shoulder arthroplasty, encompassing total shoulder arthroplasty (TSA), reverse TSA (RTSA), and hemiarthroplasty (HA). RTSA differs biomechanically from TSA and HA by altering the center of rotation and relying more heavily on deltoid function, which changes stress distribution along the humeral shaft [1]. These fractures occur in the proximal or diaphyseal humerus adjacent to the prosthetic stem and are associated with significant morbidity, including pain, functional impairment, and prolonged rehabilitation. Their management is complex due to the presence of the prosthesis, variable bone quality, and patient comorbidities. Consequently, periprosthetic humeral fractures represent a unique subset of orthopedic injuries that require careful evaluation and individualized treatment planning [2].

The incidence of periprosthetic humeral fractures after shoulder arthroplasty ranges between 0.5% and 3%, depending on the type of arthroplasty and follow-up duration [3]. The risk increases with age, female sex, osteoporosis, rheumatoid arthritis, and prior trauma. RTSA has been associated with a slightly higher fracture risk than anatomic TSA because of altered biomechanics and stress distribution along the humeral shaft. Although rare, these fractures are expected to become more common with the increasing number of shoulder arthroplasties performed globally and the aging population with osteoporotic bone.

Etiologically, periprosthetic humeral fractures typically result from low-energy trauma in osteoporotic bone or from intraoperative technical factors, including excessive humeral stem insertion force or stress risers at the tip of the prosthesis [4]. Pathophysiologically, stress concentration around the stem, poor bone stock, and preexisting bone deformities contribute to fracture susceptibility. Management strategies are dictated by stem stability, fracture pattern, and patient-specific factors. Surgical options include open reduction and internal fixation (ORIF) with plates, cables, or screws, or revision arthroplasty with long-stem prostheses, whereas nonoperative management is reserved for minimally displaced or tuberosity fractures with a stable stem [5].

Fracture classification systems, including the Cofield and Worland classifications, guide treatment planning by delineating fracture location and stem stability [6,7]. Type A (tuberosity fractures), type B (around the stem), and type C (distal to the stem) fractures in the Cofield classification and Worland B1 (spiral fracture around a stable stem), B2 (transverse fracture around a stable stem), and B3 (fracture around a loose stem) fracture patterns help stratify patients for operative versus nonoperative management [8]. The primary aim of this review is to summarize contemporary evidence on the management and outcomes of periprosthetic proximal humeral fractures following shoulder arthroplasty. The secondary aim is to evaluate factors influencing union rates, functional recovery, and complications, thereby providing a framework for evidence-based decision-making in clinical practice.

## Review

Materials and methods

Search Strategy

A systematic literature search was conducted in accordance with the PRISMA 2020 guidelines to identify studies reporting periprosthetic humeral fractures following shoulder arthroplasty [9]. The databases searched included PubMed, Embase, Scopus, and the Cochrane Library, and the search was conducted between August 1, 2025, and January 15, 2026. The search strategy combined Medical Subject Headings (MeSH) terms and free-text keywords related to the population, intervention, and outcomes of interest, using Boolean operators (AND, OR) to combine terms. Key search terms included "periprosthetic humeral fracture" OR "periprosthetic fracture humerus", "shoulder arthroplasty" OR "total shoulder arthroplasty" OR "reverse shoulder arthroplasty" OR "hemiarthroplasty", "ORIF" OR "open reduction internal fixation" OR "revision arthroplasty", and "fracture management" OR "treatment outcomes" (Table 1). Searches were conducted independently by two reviewers, and all retrieved records were imported into a reference manager, with duplicates removed before screening. Titles and abstracts were then screened against predefined eligibility criteria, followed by full-text assessment. Any disagreements between reviewers were resolved through discussion with a third reviewer.

**Table 1:** Detailed search strategy ORIF, open reduction and internal fixation

Database	Search terms	Search period	Search fields	Records identified
PubMed	("periprosthetic humeral fracture" OR "periprosthetic fracture humerus") AND ("shoulder arthroplasty" OR "total shoulder arthroplasty" OR "reverse shoulder arthroplasty" OR "hemiarthroplasty") AND ("ORIF" OR "open reduction internal fixation" OR "revision arthroplasty") AND ("fracture management" OR "treatment outcomes")	August 1, 2025 to January 15, 2026	Title/Abstract; MeSH, where applicable	n = 45
Embase	('periprosthetic humeral fracture' OR 'periprosthetic fracture humerus') AND ('shoulder arthroplasty' OR 'total shoulder arthroplasty' OR 'reverse shoulder arthroplasty' OR 'hemiarthroplasty') AND ('ORIF' OR 'open reduction internal fixation' OR 'revision arthroplasty') AND ('fracture management' OR 'treatment outcomes')	August 1, 2025 to January 15, 2026	Title/Abstract; Emtree terms	n = 38
Scopus	TITLE-ABS-KEY("periprosthetic humeral fracture" OR "periprosthetic fracture humerus") AND TITLE-ABS-KEY("shoulder arthroplasty" OR "total shoulder arthroplasty" OR "reverse shoulder arthroplasty" OR "hemiarthroplasty") AND TITLE-ABS-KEY("ORIF" OR "revision arthroplasty") AND TITLE-ABS-KEY("fracture management")	August 1, 2025 to January 15, 2026	TITLE-ABS-KEY	n = 32
Cochrane Library	("periprosthetic humeral fracture" OR "periprosthetic fracture humerus") AND ("shoulder arthroplasty" OR "total shoulder arthroplasty" OR "reverse shoulder arthroplasty" OR "hemiarthroplasty") AND ("ORIF" OR "revision arthroplasty")	August 1, 2025 to January 15, 2026	Title/Abstract/Keywords	n = 22

Eligibility Criteria

Eligibility criteria for this systematic review were defined using the PICO framework [10]. The population included adult patients (≥18 years) with periprosthetic proximal humeral fractures following shoulder arthroplasty, including TSA, RTSA, and HA, after both primary and revision procedures. Interventions of interest included surgical management, such as ORIF with plates, cables, screws, or other fixation devices, and revision arthroplasty using long-stem prostheses. Comparators included nonoperative management with slings, braces, or other conservative measures, as well as comparisons between different surgical techniques. Primary outcomes were fracture union and complication rates, including malunion, nonunion, infection, and prosthesis loosening. Secondary outcomes included pain (Visual Analog Scale, VAS), functional recovery (American Shoulder and Elbow Surgeons (ASES) [11] and Single Assessment Numeric Evaluation (SANE) scores [12]), time to return to activity, and the need for secondary procedures. Studies were included if they were original clinical studies (retrospective or prospective cohort studies, observational studies, or case series with ≥5 patients) reporting fracture type, management strategy, and outcomes, published in English, and with a minimum follow-up of 12 months to allow adequate assessment of union, nonunion, or component loosening. Exclusion criteria included case reports, animal studies, in vitro studies, conference abstracts, editorials, reviews without original data, and studies not reporting management outcomes.

Study Selection

After duplicates were removed, all retrieved records were screened independently by two reviewers based on titles and abstracts against the predefined eligibility criteria. Full texts of potentially relevant studies were then assessed for inclusion. Discrepancies were resolved through discussion with a third reviewer. The study selection process was documented using the PRISMA 2020 flow diagram, including the numbers of records identified, screened, excluded, and ultimately included in the review.

Data Extraction

Data from the included studies were extracted independently by two reviewers using a standardized data extraction form. Extracted information included study characteristics (authors, year, country, study design, and sample size), patient demographics (age, sex, and comorbidities), fracture characteristics (location and classification type), management strategy (nonoperative, ORIF, or revision arthroplasty), and outcomes (fracture union, complications, pain scores, and functional scores). Any disagreements during data extraction were resolved by consensus with a third reviewer to ensure accuracy.

Risk of Bias Assessment

The risk of bias of the included studies was assessed independently by two reviewers using validated tools according to study type. The Newcastle-Ottawa Scale (NOS) was used for cohort studies [13], the Joanna Briggs Institute (JBI) Critical Appraisal Tool for case series [14], and A MeaSurement Tool to Assess Systematic Reviews (AMSTAR 2) for systematic reviews [15]. Domains assessed included selection bias, reporting bias, measurement bias, confounding, and heterogeneity. Studies were categorized as having a low, moderate, or high risk of bias based on the criteria of each tool.

Data Synthesis

Given the heterogeneity in study design, fracture classification, and reported outcomes, a narrative synthesis of the data was performed. Quantitative outcomes, such as union rates, complication rates, pain scores, and functional scores, were summarized using descriptive statistics. Subgroup analyses were reported when possible, stratified by fracture type (Cofield or Worland classification [6,7]), stem stability, and management strategy. Meta-analysis was performed only when sufficient homogeneity in outcomes and study design was present. The synthesis aimed to identify patterns in management outcomes and factors influencing fracture healing, function, and complications.

Results

Study Selection Process

**Figure 1:**
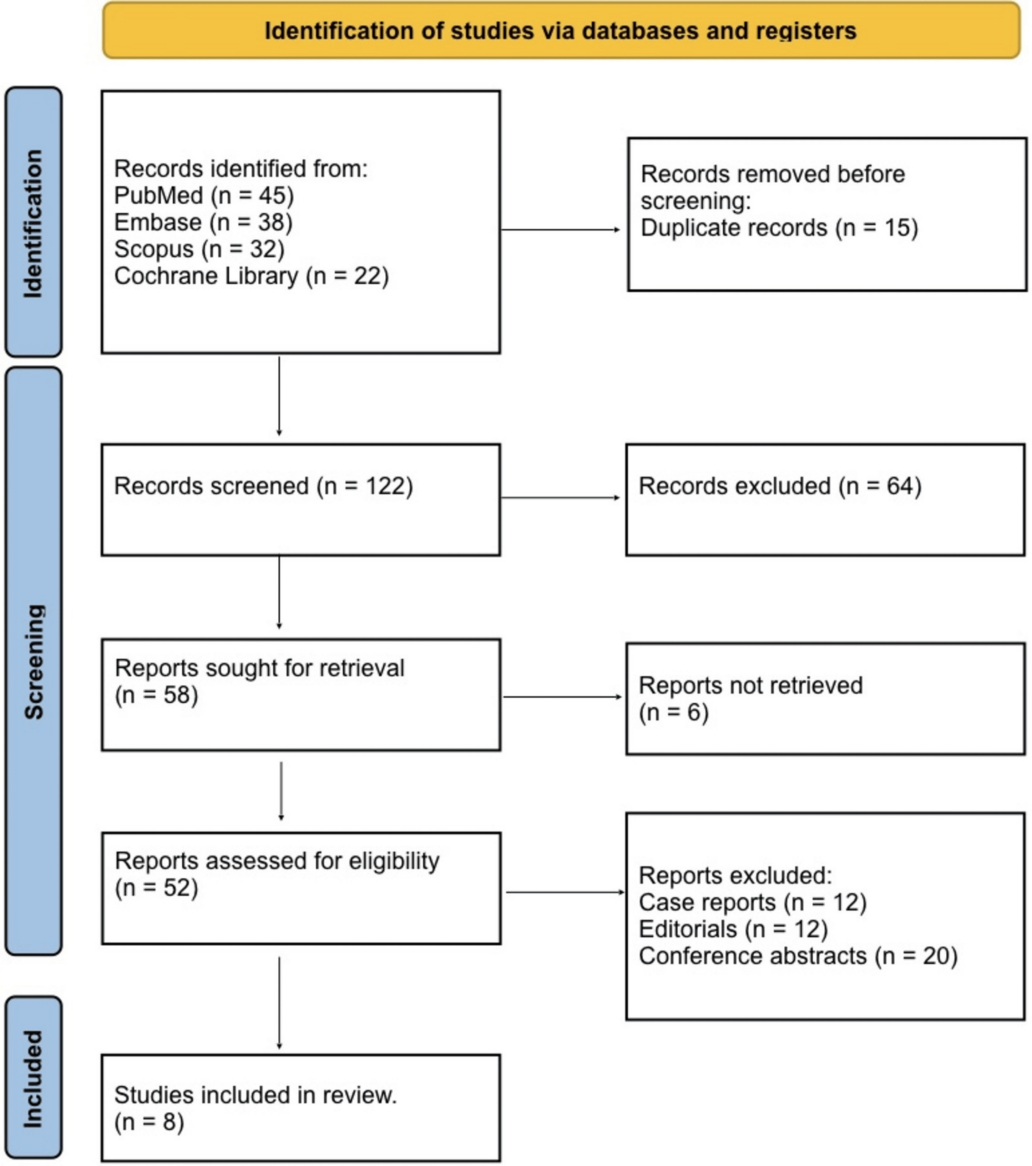
PRISMA 2020 flow diagram

Figure 1 shows the study selection process. A total of 137 records were identified through database searching, including PubMed (45 records), Embase (38 records), Scopus (32 records), and the Cochrane Library (22 records). Before screening, 15 duplicate records were removed. Titles and abstracts of the remaining 122 records were screened against the predefined eligibility criteria, resulting in the exclusion of 64 records that did not meet the inclusion criteria. Full-text articles were sought for 58 reports, of which six were not retrieved. The full texts of the remaining 52 reports were assessed for eligibility, and 44 studies were excluded for the following reasons: case reports (12), editorials (12), and conference abstracts (20). Ultimately, eight studies were included in the systematic review. The entire selection process is summarized in the PRISMA 2020 flow diagram to ensure transparency and reproducibility of the search strategy and study selection.

Characteristics of the Selected Studies

Table 2 summarizes the eight studies reporting the management of periprosthetic humeral fractures following shoulder arthroplasty [16-23]. Sample sizes ranged from 16 patients in Kumar et al. [16] to 210 patients in the pooled review by Mourkus et al. [23], including both primary and revision TSA, RTSA, and HA cases. Management strategies included nonoperative treatment, ORIF using plates, cables, or screws, and revision arthroplasty with long-stem prostheses, with treatment choice largely guided by fracture type and humeral stem stability [16-23]. Fracture patterns were reported using the Cofield (Types A-C) and Worland (Types B1-B3) classification systems, with fractures around the stem (Cofield Type B; Worland Types B1-B3) being the most common [16-18,20,22]. Outcomes assessed included union rates, pain (VAS), functional scores (ASES and SANE), and complications, with ORIF generally achieving the highest union rates and nonoperative management being associated with higher rates of nonunion or malunion [16,19,20,23]. Risk factors such as osteoporosis, female sex, rheumatoid arthritis, and low bone stock were highlighted, and the studies consistently reported that successful outcomes depended on stem stability, fracture displacement, and appropriate fracture-specific management [16,23].

**Table 2:** Characteristics of selected studies on the management of periprosthetic proximal humeral fractures following shoulder arthroplasty Cofield classification: Fracture classification for periprosthetic humeral fractures following shoulder arthroplasty: Type A, fracture of the tuberosity; Type B, fracture around the stem; Type C, fracture distal to the stem [6] Worland classification: Alternative classification of periprosthetic humeral fractures based on fracture location and stem stability: B1, spiral fracture around a stable stem; B2, transverse fracture around a stable stem; B3, fracture around a loose stem [7] ASES, American Shoulder and Elbow Surgeons; HA, hemiarthroplasty; ORIF, open reduction and internal fixation; RTSA, reverse total shoulder arthroplasty; SANE, Single Assessment Numeric Evaluation; TSA, total shoulder arthroplasty; VAS, Visual Analog Scale

Authors and year	Population	Exposure/condition	Comparator	Pathophysiological findings	Anatomical impact	Management technique	Pain	Function	Outcomes
Kumar et al. (2004) [16]	16 patients with postoperative periprosthetic humeral fractures following TSA	Periprosthetic humeral fractures occurring after TSA	Nonoperative treatment vs. ORIF vs. long-stem prosthetic revision	Stress concentration at the tip of the humeral stem; osteoporosis common; trauma-related fractures	Mostly Cofield Type B and Type C fractures occurring at or distal to the humeral stem tip	Nonoperative management using a brace (5 patients); ORIF using plates and cables (6 patients); long-stem revision prosthesis (5 patients)	Not reported numerically	Functional decline compared with the pre-fracture baseline	Union achieved in 14 of 16 patients; revision required in cases with a loose stem; highlighted the importance of stem stability in management
Steinmann and Cheung (2008) [17]	Clinical cases of periprosthetic humeral fractures following shoulder arthroplasty	Periprosthetic humeral fractures	Conservative treatment vs. surgical treatment	Implant acts as a stress riser; poor bone stock contributes to fracture	Fractures located around the humeral stem and along the diaphysis	Nonoperative management; ORIF using locking plates and cables; long-stem revision arthroplasty	Not quantified	Not quantified	ORIF was successful when the humeral stem was stable; revision was required for fractures associated with loose prosthetic components
Sanchez-Sotelo and Athwal (2022) [18]	Contemporary clinical series of postoperative periprosthetic humeral fractures following shoulder arthroplasty	Periprosthetic humeral fractures occurring after shoulder arthroplasty	ORIF vs. revision arthroplasty vs. nonoperative management	Osteopenia common; fracture type strongly associated with humeral stem stability	Type A fractures involve the tuberosity, Type B fractures occur around the stem, and Type C fractures occur distal to the stem	Nonoperative management for Type A fractures; ORIF for Type B fractures with a stable stem; revision arthroplasty for Type B fractures with a loose stem and Type C fractures	Not reported	Not reported	Fracture type-directed management resulted in improved union rates and reduced complications
Novi et al. (2021) [19]	18 elderly patients with postoperative periprosthetic humeral fractures following shoulder arthroplasty	Periprosthetic humeral fractures in osteoporotic bone	Operative vs. nonoperative treatment	Low-energy trauma; osteoporotic bone contributes to fracture risk	Fractures occurred around the prosthetic stem	ORIF using plates and cables (12 patients); nonoperative management with a sling or brace (6 patients)	Mild residual pain reported in 3 patients	Acceptable shoulder function in fractures that achieved union	Union achieved in 16 of 18 patients; ORIF resulted in faster recovery than nonoperative management
Cox et al. (2024) [20]	46 patients with postoperative periprosthetic humeral fractures following shoulder arthroplasty	Fractures classified according to Worland B1, B2, B3, and Type C patterns	Nonoperative management vs. ORIF with or without stem revision	Stable vs. unstable humeral stem patterns; osteopenia common	Fractures occurred around the humeral stem and distal humeral shaft	Nonoperative management in 18 patients; ORIF in 28 patients, including 8 patients who required stem revision	Mean VAS pain score: 2.2	ASES score: 73.5; SANE score: 66.5	High union rate; 6 of 18 patients initially treated nonoperatively crossed over to surgery; satisfactory outcomes achieved when stem stability was respected
Otworowski et al. (2023) [21]	Data pooled from 70 studies of periprosthetic humeral fractures following TSA, RTSA, and HA	Periprosthetic humeral fractures	Conservative vs. operative management	Risk factors included rheumatoid arthritis, female sex, and low body mass index; humeral stem stability was the central determinant of management	Fractures most commonly involved the humeral shaft around the prosthetic stem	Nonoperative management; ORIF; revision arthroplasty using a long-stem prosthesis	Variable among included studies	Variable among included studies	Successful fracture union occurred when management decisions were guided by humeral stem stability and fracture displacement
Tansey et al. (2024) [22]	Meta-analysis of 196 postoperative periprosthetic humeral fractures from 20 clinical studies	Periprosthetic humeral fractures following shoulder arthroplasty	ORIF vs. revision arthroplasty vs. nonoperative management	Forty-two percent of fractures were Cofield Type B; the mean time from arthroplasty to fracture was 2.9 years	Fractures occurred around the humeral stem and along the shaft	ORIF was the predominant treatment; revision arthroplasty and nonoperative treatment were used less frequently	Pain not consistently reported	Functional outcomes measured using multiple scoring systems	ORIF had the highest fracture union rate and fewer complications; revision arthroplasty had more complications; nonoperative treatment was associated with the highest malunion and treatment failure rates
Mourkus et al. (2022) [23]	210 patients from 40 studies with periprosthetic humeral fractures	Periprosthetic humeral fractures associated with shoulder or elbow arthroplasty	Fixation vs. revision arthroplasty vs. nonoperative management	Heterogeneous fracture patterns; nonoperative management resulted in poor outcomes	Fractures involved the proximal and diaphyseal humerus	Nonoperative management; ORIF; revision arthroplasty with humeral stem replacement	Mixed among included studies	Mixed among included studies	Nonoperative treatment resulted in approximately 50% nonunion; ORIF resulted in approximately 93% union; revision arthroplasty resulted in approximately 84% union, with a 29% complication rate

Risk of Bias Assessment

The risk of bias of the included studies was assessed using validated tools. The retrospective cohort studies by Kumar et al. [16], Novi et al. [19], and Cox et al. [20] were evaluated using the NOS, demonstrating a moderate risk of bias in Kumar et al. [16], a low-to-moderate risk in Novi et al. [19], and a low risk in Cox et al. [20] because of adequate sample sizes and standardized outcome reporting. The case series by Steinmann and Cheung [17] and Sanchez-Sotelo and Athwal [18] were appraised using the JBI Critical Appraisal Tool, with a high risk of bias in Steinmann and Cheung [17] and a moderate risk in Sanchez-Sotelo and Athwal [18]. The systematic reviews by Otworowski et al. [21], Tansey et al. [22], and Mourkus et al. [23] were assessed using AMSTAR 2, demonstrating a moderate risk of bias in Otworowski et al. [21] and Mourkus et al. [23] and a low-to-moderate risk in Tansey et al. [22]. Overall, study quality varied but was considered sufficient to inform management decisions (Table 3).

**Table 3:** Risk of bias assessment of included studies AMSTAR 2, A MeaSurement Tool to Assess Systematic Reviews (used for systematic reviews and meta-analyses); JBI, Joanna Briggs Institute; NOS, Newcastle-Ottawa Scale

Study	Study design	Risk of bias tool	Types of bias assessed	Risk of bias rating	Justification
Kumar et al. (2004) [16]	Retrospective cohort	NOS	Selection bias, reporting bias, confounding bias	Moderate	Small sample (16 patients); clear inclusion criteria; retrospective design limited control of confounding; outcomes reported, but functional measures were partially subjective.
Steinmann and Cheung (2008) [17]	Case series	JBI Critical Appraisal Tool	Selection bias, reporting bias	High	Limited sample size; no comparator group; outcomes were descriptive; retrospective design; incomplete quantification of pain and functional outcomes.
Sanchez-Sotelo and Athwal (2022) [18]	Retrospective case series	JBI Critical Appraisal Tool	Selection bias, reporting bias, measurement bias	Moderate	Well-described fracture classification and management; larger case series; limited numerical outcome reporting; retrospective design.
Novi et al. (2021) [19]	Retrospective cohort	NOS	Selection bias, attrition bias, reporting bias	Low to moderate	Clear inclusion and exclusion criteria; objective assessment of fracture union; small sample (18 patients); complete follow-up; some functional outcomes were subjective.
Cox et al. (2024) [20]	Retrospective cohort	NOS	Selection bias, reporting bias, performance bias	Low	Adequate sample size (46 patients); clear comparator groups; standardized functional and pain outcome measures; minor crossover in the nonoperative group.
Otworowski et al. (2023) [21]	Systematic review	AMSTAR 2	Publication bias, selection bias, reporting bias	Moderate	Comprehensive search (70 studies); substantial heterogeneity; pooled data presented; quality of the included studies varied, and risk of bias was not consistently addressed.
Tansey et al. (2024) [22]	Systematic review and meta-analysis	AMSTAR 2	Publication bias, selection bias, reporting bias, heterogeneity	Low to moderate	Included 20 studies; meta-analysis performed; moderate heterogeneity; risk of bias of the included studies was assessed; outcomes were inconsistent across some studies.
Mourkus et al. (2022) [23]	Systematic review	AMSTAR 2	Publication bias, selection bias, reporting bias, heterogeneity	Moderate	Large pooled dataset (210 patients from 40 studies); heterogeneous study designs; outcomes extracted appropriately; risk of bias of the included studies varied.

Discussion

Periprosthetic proximal humeral fractures following shoulder arthroplasty remain a complex and challenging complication, with management strategies primarily dictated by fracture type, humeral stem stability, and patient-specific factors. Surgical management, including ORIF and revision arthroplasty with long-stem prostheses, is preferred for displaced fractures or fractures around a loose stem, whereas nonoperative management is generally reserved for minimally displaced tuberosity fractures with a stable prosthesis [16,23]. Across the included studies, ORIF was the most commonly reported surgical technique, using plates, cables, or screws to restore alignment and stability while preserving the humeral stem when feasible [16,18,20,22]. Revision arthroplasty was selectively employed in cases of stem loosening or severe bone loss, highlighting the importance of individualized treatment planning based on fracture classification systems such as Cofield and Worland [16,20,23].

Outcomes across studies demonstrated that successful fracture union and functional recovery were closely associated with appropriate fracture-specific management and stem stability. ORIF generally achieved the highest union rates, ranging from approximately 88-93% across studies, with acceptable pain and functional scores, whereas nonoperative management was associated with higher rates of nonunion or malunion, particularly in displaced or unstable fractures [16,19,20,23]. Revision arthroplasty achieved moderate union rates (~84%) but carried a higher risk of complications, including infection, prosthesis loosening, and impaired function [23]. Functional outcomes, as measured by the ASES score and the SANE, were often better in patients treated surgically with stable stem fixation, reflecting the critical role of biomechanical restoration and early mobilization in rehabilitation [20,22]. Pain outcomes were reported inconsistently, although residual pain was generally mild in successfully united fractures [19,20].

Comparison across studies highlighted variability in patient populations, fracture patterns, and management approaches. Smaller retrospective cohort studies and case series (e.g., [16,17]) reported good outcomes with carefully selected ORIF or nonoperative strategies but were limited by small sample sizes and descriptive reporting. Larger pooled reviews and meta-analyses [21-23] provided broader evidence of the association between stem stability, fracture displacement, and outcomes, although heterogeneity in study design, fracture classification, and outcome measures limited direct comparisons. Across all studies, osteoporosis, female sex, rheumatoid arthritis, and low bone stock were consistently reported as risk factors influencing fracture occurrence and healing, underscoring the need for preoperative risk stratification and optimization of bone quality.

Despite these findings, several limitations exist within the current literature. Most studies were retrospective, single-center, and involved small sample sizes, introducing potential selection and reporting bias [16,20]. Heterogeneity in fracture classification, surgical technique, rehabilitation protocols, and outcome measurement hindered the ability to perform a meta-analysis and to generalize the results [21,23]. Future research should focus on prospective, multicenter studies with standardized classification systems, uniform surgical and rehabilitation protocols, and consistent reporting of pain, function, and complications. Additionally, emerging techniques, such as patient-specific instrumentation, novel fixation devices, and augmented stem designs, should be evaluated to optimize union rates and functional outcomes in this challenging patient population.

## Conclusions

Periprosthetic proximal humeral fractures following shoulder arthroplasty are uncommon but pose significant challenges because of variable fracture patterns, stem stability, and patient-specific factors. Management should be individualized, with ORIF preferred for displaced fractures with a stable stem, revision arthroplasty for loose stems or severe bone loss, and nonoperative treatment reserved for minimally displaced fractures. Successful outcomes, including fracture union and functional recovery, are closely linked to stem stability, appropriate fracture-specific management, and patient optimization. Current evidence highlights the need for standardized reporting, prospective studies, and evaluation of novel fixation techniques to guide evidence-based decision-making and improve clinical outcomes in this complex patient population.
